# Histone acetyltransferase Kat2a regulates ferroptosis via enhancing Tfrc and Hmox1 expression in diabetic cardiomyopathy

**DOI:** 10.1038/s41419-024-06771-x

**Published:** 2024-06-10

**Authors:** Juan Zhen, Xia Sheng, Tianlong Chen, Haitao Yu

**Affiliations:** 1 Department of Cadre Ward, the First Hospital of Jilin University, Changchun, 130021 Jilin China; 2 Department of Cardiology, the First Hospital of Jilin University, Changchun, 130021 Jilin China

**Keywords:** Mechanisms of disease, Cardiomyopathies, Cell death

## Abstract

Diabetic cardiomyopathy (DCM) is a prevalent myocardial microvascular complication of the myocardium with a complex pathogenesis. Investigating the pathogenesis of DCM can significantly contribute to enhancing its prevention and treatment strategies. Our study revealed an upregulation of lysine acetyltransferase 2 A (Kat2a) expression in DCM, accompanied by a decrease in N6-methyladenosine (m6A) modified Kat2a mRNA levels. Our study revealed an upregulation of lysine acetyltransferase 2 A (Kat2a) expression in DCM, accompanied by a decrease in N6-methyladenosine (m6A) modified Kat2a mRNA levels. Functionally, inhibition of Kat2a effectively ameliorated high glucose-induced cardiomyocyte injury both in vitro and in vivo by suppressing ferroptosis. Mechanistically, Demethylase alkB homolog 5 (Alkbh5) was found to reduce m6A methylation levels on Kat2a mRNA, leading to its upregulation. YTH domain family 2 (Ythdf2) played a crucial role as an m6A reader protein mediating the degradation of Kat2a mRNA. Furthermore, Kat2a promoted ferroptosis by increasing Tfrc and Hmox1 expression via enhancing the enrichment of H3K27ac and H3K9ac on their promoter regions. In conclusion, our findings unveil a novel role for the Kat2a-ferroptosis axis in DCM pathogenesis, providing valuable insights for potential clinical interventions.

## Introduction

Diabetic cardiomyopathy (DCM) is a prevalent microvascular complication affecting the myocardium and constitutes the primary cause of mortality among diabetic patients [1]. It can result in cardiac insufficiency, myocardial fibrosis, myocardial hypertrophy, and heart failure [2]. The indistinct clinical features observed during early-stage DCM pose diagnostic challenges [3]. The pathogenesis of DCM involves intricate and multifactorial mechanisms including metabolic disorders, mitochondrial dysfunction, oxidative stress, inflammation, and cell death [4]. Currently, the clinical management of DCM poses significant challenges due to the absence of specific treatment strategies [1]. Therefore, it is imperative to further elucidate the underlying mechanisms driving DCM pathogenesis.

Emerging evidence suggests that abnormal gene expression resulting from coordinated regulation involving transcriptional and epigenetic factors, such as histone modifications and DNA alterations, may play a pivotal role in cardiovascular diseases [5]. Lysine acetyltransferase 2 A (KAT2A), also known as GCN5, enhances target gene transcription by augmenting histone acetylation modifications [6]. Kat2a has been implicated in intracellular lipid accumulation in Arrhythmogenic cardiomyopathy [7]. Additionally, Kat2a has been reported to promote pathological cardiac hypertrophy and modulate cardiac metabolism in heart failure cases [8]. However, the involvement of Kat2a in DCM remains undisclosed; thus this study aims to investigate its role along with potential regulatory mechanisms.

Multiple cell death modes, including apoptosis, ferroptosis, autophagy, pyroptosis, and necroptosis, are involved in the development of DCM [9]. Ferroptosis, an iron-dependent form of regulatory cell death, is activated in DCM and has been shown to play an indispensable role in its pathogenesis [10]. The inhibition of cardiomyocyte ferroptosis could alleviate the progression of DCM [11, 12]. Ferroptosis is implicated in myocardial injury in type 2 diabetes mellitus (T2DM), and inhibition of retinol dehydrogenase 10 (RDH10) promotes retinol metabolism disorder, thereby promoting ferroptosis in the heart [13]. Sulforaphane inhibits ferroptosis and its associated pathogenesis in DCM through AMPK-mediated NRF2 activation [10]. Zhang et al. demonstrate that autophagy initiates NrF2-mediated ferroptosis in cardiomyocytes, thereby exacerbating the progression of DCM [14]. However, our understanding of the complex pathway involved in ferroptotic mechanisms underlying DCM remains limited. Therefore, we aimed to further elucidate the role of ferroptosis in DCM and its underlying regulatory mechanisms.

In this study, we have demonstrated that Kat2a expression is enhanced in mice with DCM, and cardiac reduction of Kat2a ameliorates DCM by inhibiting ferroptosis through epigenetic silencing of Tfrc and Hmox1 transcription.

## Materials and methods

### Dataset and Identification of DEGs

The gene expression profiles of heart samples for GSE173384 and GSE161931 were obtained from the Gene Expression Omnibus (GEO) database (https://www.ncbi.nlm.nih.gov/geo/). For GSE173384, MeRIP-seq and RNA-seq analyses were performed to investigate the m6A patterns in dilated cardiomyopathy (DCM) and normal hearts. This dataset included heart samples from five control mice and five DCM mice. For GSE161931, heart samples from 5 db/db T2D mice and 5 BKS mice were included to identify differentially expressed genes associated with DCM. Differential expression analysis was conducted by applying a fold change threshold of > 2 or < 0.5 along with a significance level of P < 0.05 to filter out differently expressed genes (DEGs) between DCM hearts and normal hearts. To identify aberrantly expressed M6A-modified genes in GSE173384, we applied a cutoff criterion of FC > 1.5 or < 1/1.5 combined with P < 0.05.

### Animals model

All animal experiments were approved by the Ethics Committee of the First Hospital of Jilin University and complied with the Guide for the Care and Use of Laboratory Animals published by the US National Institutes of Health. C57BL/6 mice (male and aged 6–8 weeks) were purchased from GemPharmatech (Nanjing, Jiangsu, China). DCM mice were established as reported by Wang et al. [10]. Mice were randomly subjected to a high-fat diet (HFD) for 12 weeks, followed by a single intraperitoneal injection of streptozotocin (STZ) (dissolved in 0.1 mol/L of citrate acid buffer, pH 4.5, 75 mg/kg) to induce hyperglycemia. Seven days after STZ injection, blood glucose levels were assessed via tail vein sampling. Mice exhibiting fasting blood glucose levels exceeding 220 mg/dL were classified as diabetic, while age-matched mice that consumed a standard diet served as the control group. For cardiomyocyte-specific Kat2a knockdown in vivo, a total of 100 μl (5.0 × 10^13^ VG/ml) adeno-associated virus 9 (AAV9) carrying shRNA against Kat2a (AAV-shKat2a) was randomly applied per mouse via tail vein injection. AAV9 carrying scramble shRNA (AAV-shNC) was used as control. Cardiac function was assessed four weeks following the AAV injection, and the heart tissues were then isolated.

### Cell culture and treatment

Primary neonatal mouse ventricular myocytes (NMVMs) were isolated from C57BL/6 mouse (1–2-day-old) hearts digested with type-II collagenase as previously described [15]. The cells were cultured in Dulbecco’s Modified Eagle Medium (DMEM) supplemented with 10% fetal bovine serum (FBS) and antibiotics at 37 °C in a humidified atmosphere with 5% CO_2_. When the cardiomyocyte populations reached 50–60% confluence, cells were exposed to glucose at a concentration of 5.5 mmol/L (normal control) or 50 mmol/L (high glucose, HG) for 48 h to imitate hyperglycemic conditions. Gene overexpression or knockdown was achieved through Adeno-associated virus 9 (AAV9) infection 6 h prior to HG treatment. Actinomycin D (Sigma-Aldrich, St. Louis, MO, USA) was used at a concentration of 5 μg/ml. For the in vitro rescue experiment, Fer-1 (2 μM) was added 1 h before HG treatment.

### Virus preparation

AAV9 encoding GFP (AAV-GFP), Alkbh5 (AAV-Alkbh5), Ythdf2 (AAV-Ythdf2), Kat2a (AAV-Kat2a), Tfrc (AAV-Trfc), Hmox1 (AAV-Hmox1), scramble shRNA (AAV-shNC), Alkbh5 shRNA (AAV-shAlkbh5), Ythdf2 shRNA (AAV-shYthdf2), Trfc shRNA (AAV-shTrfc), Hmox1 shRNA (AAV-shHmox1) and Kat2a shRNA (AAV-shKat2a) was applied to infect NMVMs in vitro. All virus was provided by Vigene Biosciences. The targeting sequence of shRNA is listed in Supplementary Table S1.

### Quantitative Real-Time RT-PCR

Total RNA was extracted from cells and heart tissues by Trizol reagent (Invitrogen). After reversed BeyoRT™ II M-MLV reverse transcriptase (Beyotime, Wuhan, China), the cDNA was amplificated by BeyoFast™ SYBR Green qPCR Mix (Beyotime). The primers are shown in Supplementary Table S2. Expression of the PCR data were shown as 2^−ΔΔct^ and Gapdh was used as an internal reference.

### Western blotting

Total protein in cells and heart tissues was extracted by RIPA lysis buffer. Then, lysates were prepared for SDS-PAGE analysis and transferred onto PVDF membranes (Merck Millipore, Billerica, MA, USA). The PVDF membranes were subsequently incubated with the primary antibodies followed by HRP-conjugated secondary antibodies. Finally, the blots were developed using enhanced chemiluminescence reagents (Beyotime). Primary antibodies for Kat2a (#3305, CST), Gapdh (#92310, CST), Fto (#45980, CST), Alkbh5 (#80283, CST), Ythdf2 (#80014, CST), Tfrc (ab214039, Abcam), Hmox1 (ab189491, Abcam), RCAN1 (ab140131, Abcam), BNP (ab19645, Abcam), ANP (ab225844, Abcam), β-MHC (MAB90961, R&D systems), Mmp2 (ab37150, Abcam), Collagen I (sc-28654, Santa Cruz Biotechnology), α-SMA (A2547, Sigma), and Tgf-β1 (ab215715, Abcam) were used.

### RNA immunoprecipitation (RIP) assay

The RNA immunoprecipitation was performed using the EZ-Magna RIP kit (Millipore, Burlington, MA, USA) following the manufacturer’s instructions. Briefly, cells and heart tissues were lysed with RIP lysis buffer. Cell extracts were co-immunoprecipitated with antibodies against m6A (ab195352, Abcam), Alkbh5 (ab234528, Abcam), and Ythdf2 (ab220163, Abcam). The precipitated RNA was subsequently purified and quantified by qRT-PCR.

### Determination of cell death

Cell death was investigated by using PI (Roche) assay. Briefly, NMVMs were digested and resuspended, followed by the addition of 2 μg/ml PI to the cell resuspension solution for a duration of 15 min. Subsequently, dead cells (PI-positive cells) were analyzed after washing.

### Lipid ROS detection

NMVMs were collected and resuspended in PBS. Then, the cells were stained with 5 μM C11-BODIPY581/591 (D3861, Thermo Fisher Scientific) for 30 min in the dark. After washing, lipid ROS were measured in a Bio-Tek fluorescence microplate reader (Winooski, VA).

### Determination of labile iron levels

Labile iron levels in NMVMs and the cardiac tissues were measured using an Iron Colorimetric Assay Kit (Cat#K390-100, BioVision, Milpitas, CA, USA), following the manufacturer’s instructions.

### Malondialdehyde (MDA) measurement

NMVMs and heart tissues were used for MDA measurement with a kit (Beyotime) according to the manufacturer’s instructions.

### GSH and oxidized GSH (GSSG) measurement

GSH and GSSG levels in NMVMs and heart tissues were measured using the Glutathione Fluorometric Assay Kit (Cat#K264-100, BioVision), according to the manufacturer’s protocol.

### Chromatin immunoprecipitation (ChIP) assay

The ChIP assay was conducted using an EZ-ChIP kit (Sigma-Aldrich) according to the manufacturer’s instructions. Briefly, after crosslinking with 1% formaldehyde, cells were lysed and subjected to sonication for 5 min. The lysates were incubated with protein A/G Sepharose preincubated with anti-Kat2a (ab217876, Abcam), H3K27ac (ab177178, Abcam), and H3K9ac (ab32129, Abcam) antibodies overnight at 4 °C. Then, the beads were washed and eluted in the elution buffer. The precipitated DNA was purified and measured by qPCR. The following primers were used in ChIP assays. Tfrc, forward: 5’-GAGGTTGTCTTAGTAAGTCA-3’; reverse: 5’-CACGCTTACATAGTCCTGTA-3’. Hmox1, forward: 5’- GATTACTAGGCATCATAAGG-3’; reverse: 5’-CTATTAATGACTACTTCTAAT -3’. The value of enrichment was calculated relative to the input and the ratio to IgG.

### Luciferase reporter assay

For luciferase assay, the Tfrc promoter (−1465-12) and Hmox1 promoter (−1562-21) were subcloned into the pGL3.0-basic vector (Promega). A total of 500 ng of each indicated plasmid was co-transfected into NMVMs. Cell lysates were harvested after 24 h transfection and measured using the dual-luciferase assay system (Promega).

### Echocardiography assessment

Mice underwent anesthesia with 1.5% isoflurane before cardiac configuration assessment using an ultrasonic diagnostic apparatus (Visual Sonic Vevo 2100, Toronto, ON, Canada) with a probe frequency of 30 MHz to obtain parasternal long-axis views. Cardiac images generated through two-dimensional M-mode echocardiography were used to calculate left ventricular ejection fraction (LVEF) and left ventricular fractional shortening (LVFS).

### Histological analysis

Heart tissues were fixed in a 4% paraformaldehyde solution and embedded in paraffin. Sequential slices were obtained from the heart tissues, which were then subjected to HE staining, Masson staining, and immunofluorescence (IF) staining. For Masson staining, Modified Masson’s Trichrome Stain Kit (G1345, Solarbio, Beijing, China) was used following the manufacturer’s instructions. For IF staining, after blocking with 3% hydrogen peroxide, the sections were subjected to heat treatment at 95 °C for 10 min using citrate buffer (Beyotime). After permeabilization with 0.3% Triton-100 and blocking with 5% BSA, the sections were then indicated with Wheat Germ Agglutinin FITC Conjugate antibody (WGA-FITC, Q-0098166, XI’AN QIYUE BIOLOGY, Xi’an, China) for 30 min. The staining of Kat2a (ab217876, Abcam) was performed using the tyramide signal amplification multiplex fluorescence staining kit (#G1236-100T, Servicebio, Wuhan, Hubei, China). Following PBS washing steps, DAPI counterstaining was applied, and the samples were visualized by a fluorescence microscope equipped with a digital camera (Axio Al, Carl Zeiss, Germany). The positive area was quantified by analyzing multiple fields per sample using the Image-pro Plus software program.

### ELISA

Cardiac interleukin IL-6 and TNF-α levels were determined using commercially available ELISA kits (R&D Systems, Inc., USA).

### Biochemical analysis

Lactate dehydrogenase (LDH), creatine kinase-MB (CK-MB), and aspartate transaminase (AST) were determined by the automatic biochemical analyzer (Toshiba Accute TBA-40FR, Japan).

### Statistical analysis

Statistical analysis was performed using GraphPad Prism 8.0 software. All values are presented as mean ± standard deviation. Two-group comparisons were analyzed using Student’s t-test, while multi-group comparisons were conducted through one-way analysis of variance (ANOVA) followed by Bonferroni post hoc analysis. Each experiment was repeated at least three times to ensure reproducibility of results.

## Results

### Kat2a is increased in DCM

**Fig. 1:**
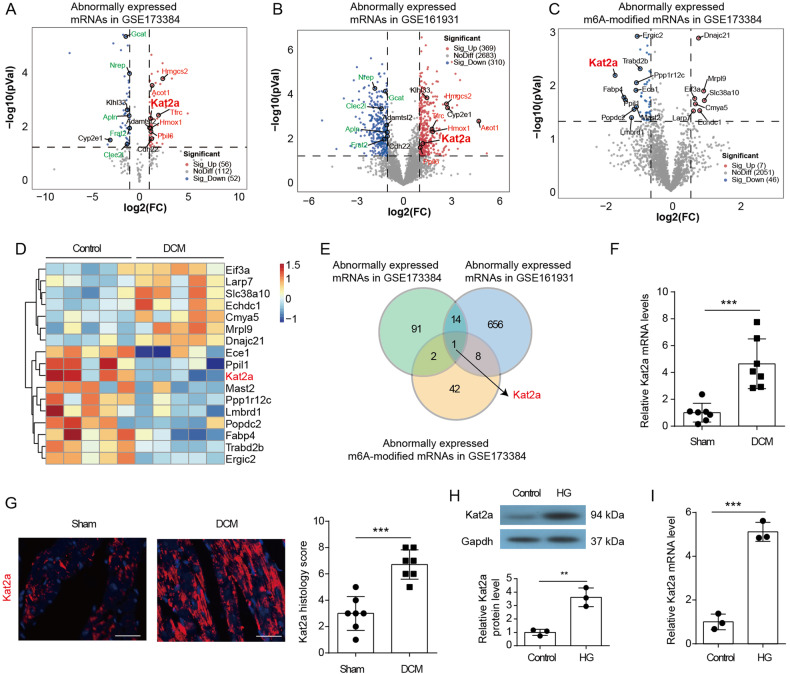
Kat2a was upregulated in DCM and HG-treated NMVCs. **A** Identification of DEGs in GSE173384. **B** Identification of DEGs in GSE161931. **C** Identification of abnormally expressed m6A-modified mRNAs in GSE173384. **D** The upregulated and top 10 downregulated m6A-modified mRNAs were shown in a heat map. **E** Kat2a mRNA was identified to be anomalously expressed in DCM along with abnormal m6A modification. **F** Kat2a mRNA expression in heart tissues of control and DCM mice (N = 7 mice/group). **G** Kat2a expression in heart tissues of control and DCM mice was investigated by IF staining (N = 7 mice/group). Scale bar = 50 μm. **H**, **I** Kat2a protein and mRNA expression in NMVCs with or without HG treatment (N = 3 independent experiments). Significance tested using Student’s t-test. Statistical significance is shown as **p < 0.01, ***p < 0.001.

To reveal the pathogenesis of DCM, we conducted a comprehensive analysis of gene expression profiles in public available datasets from NCBI-GEO, aiming to identify differentially expressed genes (DEGs). A total of 108 DEGs (56 increased and 52 decreased) were identified in GSE173384 (Fig. 1A) and 679 DEGs (369 increased and 310 decreased) were identified in GSE161931 (Fig. 1B). In addition, patterns of m6A in DCM and normal hearts were analyzed by MeRIP-seq in GSE173384. A total of 53 abnormally expressed m6A-modified mRNAs (7 increased and 46 decreased) were identified (Fig. 1C). The upregulated and top 10 downregulated m6A-modified mRNAs were shown in a heat map (Fig. 1D). Significantly, Kat2a mRNA ranked among the top 10 m6A-modified mRNAs with abnormal expression in DCM. The overlap analysis revealed Kat2a shared among these datasets (Fig. 1E), indicating that the abnormal expression of Kat2a in DCM may be caused by the abnormal m6A modification of its mRNA. By integrating the findings from our bioinformatics analysis, we revealed an upregulation of Kat2a expression in DCM, accompanied by a decrease in m6A-modified Kat2a mRNA levels. Consequently, our focus has shifted towards investigating the function of Kat2a in DCM and exploring its underlying regulatory mechanisms. We then detected the cardiac expression level of Kat2a in sham and DCM mice. Kat2a protein level and mRNA expression in cardiac tissue were significantly increased (Fig. 1F, G). Besides, an increased Kat2a level was identified in neonatal mouse ventricular cardiomyocytes (NMVCs) treated with HG when compared with control cells (Fig. 1H, I). In a word, these data indicate that Kat2a is increased in DCM and may be a key molecular regulator during DCM.

### Alkbh5-indued demethylation promotes Kat2a expression

**Fig. 2:**
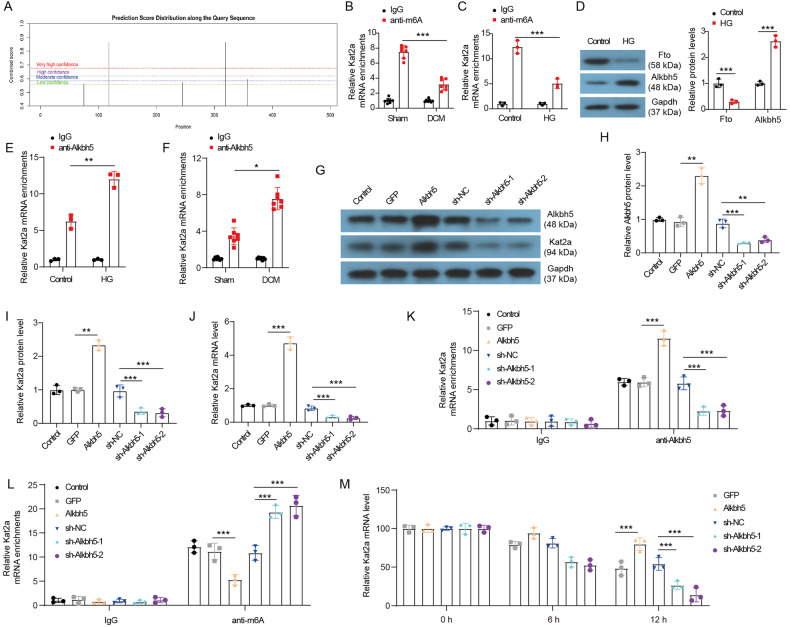
Alkbh5 promoted Kat2a expression by reducing m6A modification. **A** Predicted binding sites of m6A modification at 3’UTR of Kat2a mRNA according to the online SRAMP database (http://www.cuilab.cn/sramp). **B** Methylated Kat2a level in heart tissues of Sham and DCM mice (N = 7 mice/group). **C** Methylated Kat2a level was detected via RIP assay in NMVCs with or without HG treatment (N = 3 independent experiments). **D** Fto and Alkbh5 protein expression in NMVCs with or without HG treatment (N = 3 independent experiments). **E** The binding between Alkbh5 protein and Kat2a mRNA was evaluated by RIP assays in NMVCs with or without HG treatment (N = 3 independent experiments). **F** The binding between Alkbh5 protein and Kat2a mRNA was evaluated by RIP assays in heart tissues of Sham and DCM mice (N = 7 mice/group). **G**–**I** The changes in Alkbh5 and Kat2a protein levels in NMVCs were evaluated by western blotting (N = 3 independent experiments). **J** Kat2a mRNA level in NMVCs was evaluated by qPCR (N = 3 independent experiments). **K** The effect of Alkbh5 overexpression or knockdown on the binding between Alkbh5 protein and Kat2a mRNA (N = 3 independent experiments). **L** The effect of Alkbh5 overexpression or knockdown on the expression of methylated Kat2a mRNA (N = 3 independent experiments). **M** Alkbh5 overexpressed or depleted NMVCs were treated with ActD, and then existing Kat2a mRNA was detected at different time points (N = 3 independent experiments). Significance tested using One-way ANOVA. Statistical significance is shown as *p < 0.05, **p < 0.01, ***p < 0.001.

Previous findings suggest that m6A modification of Kat2a mRNA may affect the expression of Kat2a mRNA. Further analysis of Kat2a mRNA sequences using the SRAMP database revealed that there were multiple potential m6A modification sites in 3’UTR of Kat2a mRNA (Fig. 2A). The results of m6A RIP assays confirmed the presence of m6A modification in Kat2a mRNA in NMVCs and cardiac tissues (Fig. 2B, C). A reduction in m6A-modified Kat2a mRNA was observed in cardiac tissues of DCM mice (Fig. 2B). Consistently, a decreased level of m6A-modified Kat2a mRNA was found in NMVCs treated with HG when compared with control cells (Fig. 2C). The demethylases Fto and Alkbh5 have been identified as m6A erasers proteins capable of removing m6A modifications from RNAs [16]. We further explored whether the decrease in m6A-modified Kat2a mRNA observed in DCM was attributed to Fto or Alkbh5. HG treatment significantly reduced Fto protein expression and upregulated Alkbh5 protein expression in NMVCs (Fig. 2D). Subsequently, we focused on elucidating the regulatory role of Alkbh5 in the m6A modification and expression of Kat2a. The RIP assay verified the binding between Alkbh5 protein and Kat2a mRNA in NMVCs and cardiac tissues (Fig. 2E, F). Besides, the binding between Alkbh5 protein and Kat2a mRNA was strengthened in NMVCs treated with HG as well as in cardiac tissues in DCM mice. Alkbh5 overexpression increased Kat2a protein and mRNA expression in NMVCs, whereas Alkbh5 knockdown significantly decreased their expression in NMVCs (Fig. 2G–J). Furthermore, Alkbh5 overexpression enhanced the binding between Alkbh5 protein and Kat2a mRNA, while Alkbh5 knockdown reduced the binding between Alkbh5 protein and Kat2a mRNA (Fig. 2K). Consistently, Alkbh5 overexpression reduced the level of m6A-modified Kat2a mRNA, whereas Alkbh5 knockdown increased the level of m6A-modified Kat2a mRNA in NMVCs (Fig. 2L). Moreover, the decay rate of Kat2a mRNA was significantly slower when Alkbh5 was overexpressed in NMVCs but faster when it was depleted (Fig. 2M). Therefore, the above results display that the reduction of m6A modification of Kat2a mRNA induced by Alkbh5 contributes to its overexpression in DCM.

### Ythdf2 is essential for the degradation of m6A-modified Kat2a mRNA

**Fig. 3:**
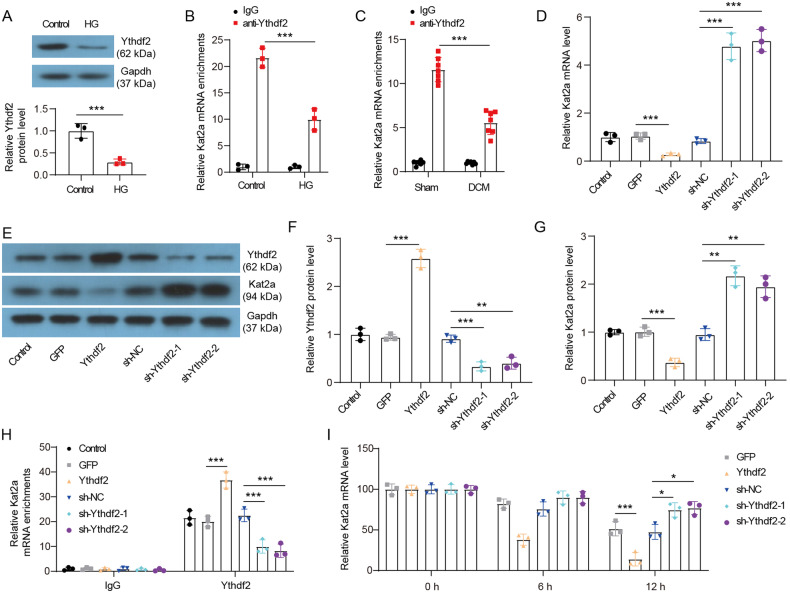
Ythdf2 promoted the degradation of Kat2a mRNA. **A** Ythdf2 protein expression in NMVCs with or without HG treatment (N = 3 independent experiments). **B** The binding between Ythdf2 protein and Kat2a mRNA was evaluated by RIP assays in NMVCs with or without HG treatment (N = 3 independent experiments). **C** The binding between Ythdf2 protein and Kat2a mRNA was evaluated by RIP assays in heart tissues of Sham and DCM mice (N = 7 mice/group). **D** Kat2a mRNA level in NMVCs with Ythdf2 overexpression or knockdown (N = 3 independent experiments). **E**–**G** The changes of Ythdf2 and Kat2a protein levels in NMVCs with Ythdf2 overexpression or knockdown (N = 3 independent experiments). **H** The effect of Ythdf2 overexpression or knockdown on the binding between Ythdf2 protein and Kat2a mRNA (N = 3 independent experiments). **I** Ythdf2 overexpressed or depleted NMVCs were treated with ActD, and then existing Kat2a mRNA was detected at different time points (N = 3 independent experiments). Significance tested using: Student’s t-test (**A**) and One-way ANOVA (**B**–**I**). Statistical significance is shown as *p < 0.05, **p < 0.01, ***p < 0.001.

The recognition of m6A-modified RNA by m6A reader proteins, such as Ythdf family, constitutes a crucial aspect of m6A methylation. Given that YTHDF2 is involved in the regulation of m6A-dependent RNA degradation [17], we hypothesized that Ythdf2 may be essential for the degradation of m6A-modified Kat2a mRNA. Notably, a reduction in Ythdf2 expression was observed in NMVCs treated with HG (Fig. 3A). The results of the RIP assay confirmed the binding between Ythdf2 protein and Kat2a mRNA in NMVCs and cardiac tissues (Fig. 3B, C). Treatment with HG decreased the binding between Ythdf2 protein and Kat2a mRNA in NMVCs, while DCM reduced this binding in cardiac tissues. Ythdf2 overexpression inhibited both Kat2a protein and mRNA expression in NMVCs, whereas Ythdf2 knockdown significantly upregulated these expression (Fig. 3D–G). Furthermore, Ythdf2 overexpression enhanced the binding between Ythdf2 protein and Kat2a mRNA, while Ythdf2 knockdown reduced this binding (Fig. 3H). Additionally, the decay rate of Kat2a mRNA was significantly slower in Ythdf2-depleted NMVCs but faster in Ythdf2-overexpressed NMVCs (Fig. 3I, J). Thus, our results indicate that Ythdf2 promotes the degradation of Kat2a mRNA.

### Kat2a enhances ferroptosis in vitro

**Fig. 4:**
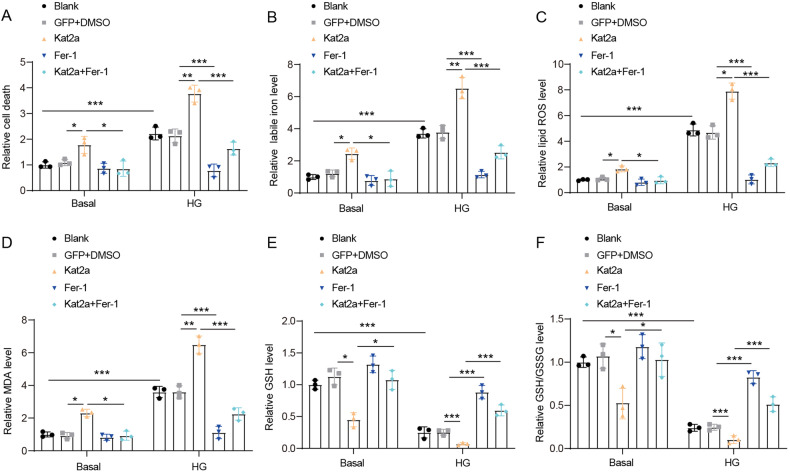
Kat2a enhanced ferroptosis of NMVCs under basal and HG conditions. **A** The cell death of NMVCs was investigated (N = 3 independent experiments). **B** The level of labile iron in NMVCs was measured (N = 3 independent experiments). **C** The level of lipid ROS in the NMVCs (N = 3 independent experiments). **D** MDA level in NMVCs (N = 3 independent experiments). **E**, **F** GSH and GSH/GSSG levels in NMVCs (N = 3 independent experiments). Significance tested using One-way ANOVA. Statistical significance is shown as *p < 0.05, **p < 0.01, ***p < 0.001.

Ferroptosis, a unique type of cell death characterized by iron overload and lipid peroxidation, plays a crucial role in the progression of DCM [10]. An increased number of dead cells were observed in NMVCs treated with HG (Fig. 4A). Increased levels of labile iron and accumulation of lipid ROS were observed in NMVCs treated with HG (Fig. 4B, C). Lipid peroxidation, a hallmark feature of ferroptosis, exhibited an increase in HG-treated NMVCs based on malondialdehyde (MDA) level assessment (Fig. 4D). Loss of GSH is another key mechanism underlying ferroptosis, and HG treatment resulted in decreased GSH level and GSH/GSSG ratio in NMVCs (Fig. 4E, F). Thus, we confirmed that HG could promote the ferroptosis of NMVCs. Under basal conditions, Kat2a overexpression promoted the death of NMVCs, increased the accumulation of labile iron, lipid ROS and MDA, while diminishing the level of GSH and GSH/GSSG. These findings indicated its potential to induce ferroptosis in NMVCs. Conversely, the inhibitor of ferroptosis, Ferrostatin-1 (Fer-1), exhibited negligible impact on cell death and labile iron, lipid ROS, MDA, GSH, GSH/GSSG levels under basal conditions. However, the alterations induced by Kat2a overexpression were effectively reversed upon Fer-1 treatment under basal conditions. Furthermore, Fer-1 treatment effectively reversed HG-induced ferroptosis. Kat2a overexpression aggravated HG-induced ferroptosis. Fer-1 partially rescued the aggravated ferroptosis caused by Kat2a overexpression under HG conditions. Collectively, these findings suggest that Kat2a regulates ferroptosis in NMVCs under both HG and basal conditions.

### Kat2a epigenetically promotes Tfrc and Hmox1 expression by enhancing H3K27ac and H3K27ac

**Fig. 5:**
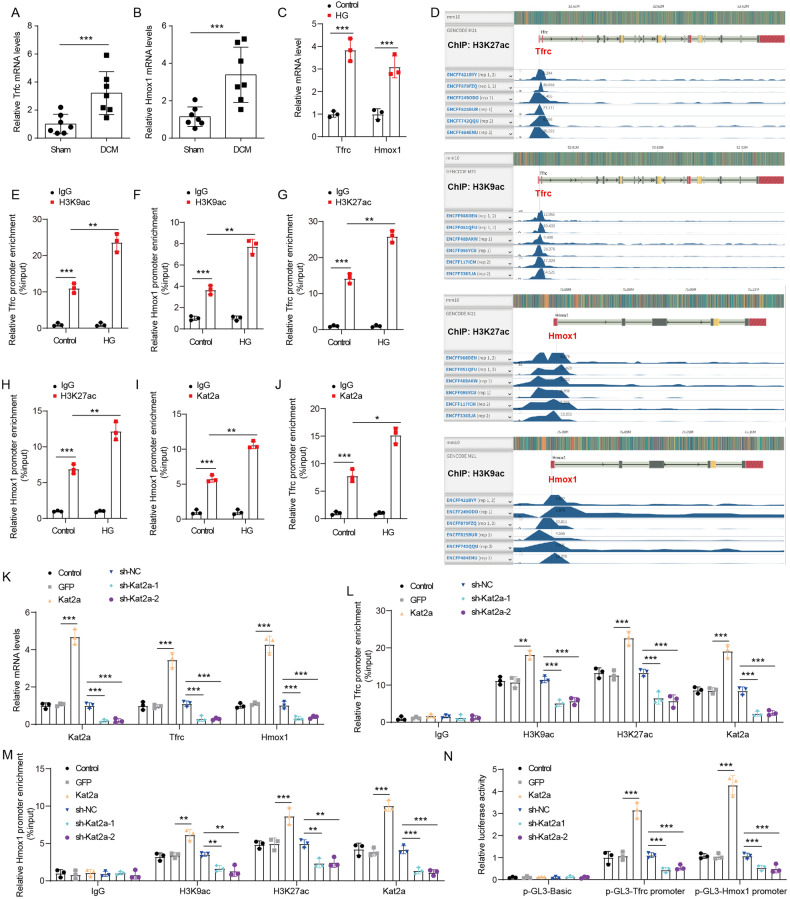
Kat2a promoted Tfrc and Hmox1 expression. **A, B** Tfrc and Hmox1 mRNA expression in heart tissues of control and DCM mice (N = 7 mice/group). **C** Tfrc and Hmox1 mRNA expression in NMVCs with or without HG treatment (N = 3 independent experiments). **D** The enrichment of H3K27ac and H3K9ac signals in the promoter region of Trfc and Hmox1 were visualized by ENCODE. **E**, **H** The enrichment of H3K27ac and H3K9ac signals in the promoter region of Trfc and Hmox1 were investigated by ChIP assay (N = 3 independent experiments). **I** The binding between Kat2a and Hmox1 promoter was verified by ChIP assay (N = 3 independent experiments). **J** The binding between Kat2a and Trfc promoter was verified by ChIP assay (N = 3 independent experiments). **K** The mRNA expression of Kat2a, Trfc, and Hmox1 in Kat2a overexpressed or depleted NMVCs (N = 3 independent experiments). **L** ChIP assay was used to assess the enrichment of H3K27ac, H3K9ac, and Kat2a in Trfc promoter in NMVCs (N = 3 independent experiments). **M** ChIP assay was used to assess the enrichment of H3K27ac, H3K9ac, and Kat2a in the Hmox1 promoter in NMVCs (N = 3 independent experiments). **N** Dual luciferase activity assays to analyze the fluorescence intensity of Kat2a-overexpressing and Kat2a-depleted NMVCs with the Trfc or Hmox1 promoter region (N = 3 independent experiments). Significance tested using: Student’s t-test (**A**, **B**) and One-way ANOVA (**C**, **E**–**N**). Statistical significance is shown as *p < 0.05, **p < 0.01, ***p < 0.001.

Ferroptosis plays an important role in the progression of DCM, and attenuation of ferroptosis can alleviate DCM-induced cardiac damage. Through bioinformatics analysis, we previously identified Trfc and Hmox1 as highly expressed ferroptosis-related genes in DCM (Fig. 1A, B). Herein, our qPCR assay results further confirmed the upregulation of Trfc and Hmox1 in DCM (Fig. 5A, B). Moreover, elevated expression of Trfc and Hmox1 were found in HG-treated NMVCs (Fig. 5C). In addition, by analyzing ChIP-seq data from cardiac tissue on ENCODE (Encyclopedia of DNA Elements) platform, an enriched signals of H3K27ac and H3K9ac were found within the promoter region of Trfc and Hmox1 (Fig. 5D), suggesting that activation of cardiac Trfc and Hmox1 may be regulated by histone acetylation modification. As a histone acetyltransferase, Kat2a has been reported to regulate gene expression by enhancing H3K27ac and H3K9ac [18]. Based on this, we speculated that Kat2a might affect the transcription and expression of Trfc and Hmox1 by regulating the histone acetylation of their promoters. The data of the ChIP assay indicated that abundant H3K27ac and H3K9ac signals were enriched in Trfc and Hmox1 promoters in NMVCs (Fig. 5E–H). Treatment with HG resulted in enhanced levels of H3K27ac and H3K9ac signals at the Trfc and Hmox1 promoters in NMVCs. Besides, we also found that Kat2a proteins could bind to the Trfc and Hmox1 promoter (Fig. 5I, J) and the binding was reinforced in NMVCs treated with HG. Kat2a overexpression significantly increased the expression of Kat2a, Trfc, and Hmox1, whereas Kat2a knockdown inhibited their expression in NMVCs (Fig. 5K). Kat2a overexpression increased the enrichment of Kat2a, H3K9ac, and H3K27ac in both Trfc and Hmox1 promoters (Fig. 5L, M), whereas Kat2a knockdown played the opposite effect. The results of the luciferase reporter assay indicated that luciferase activity in the p-GL3-promoter group was higher than in the p-GL3-Basic group. Kat2a overexpression markedly enhanced Trfc and Hmox1 promoter activity and Kat2a knockdown reduced Trfc and Hmox1 promoter activity (Fig. 5N). In summary, our study indicated that Kat2a enhances Tfrc and Hmox1 expression by promoting H3K27ac and H3K27ac in their respective promoter.

### Kat2a regulates ferroptosis via targeting Tfrc and Hmox1 in vitro

**Fig. 6:**
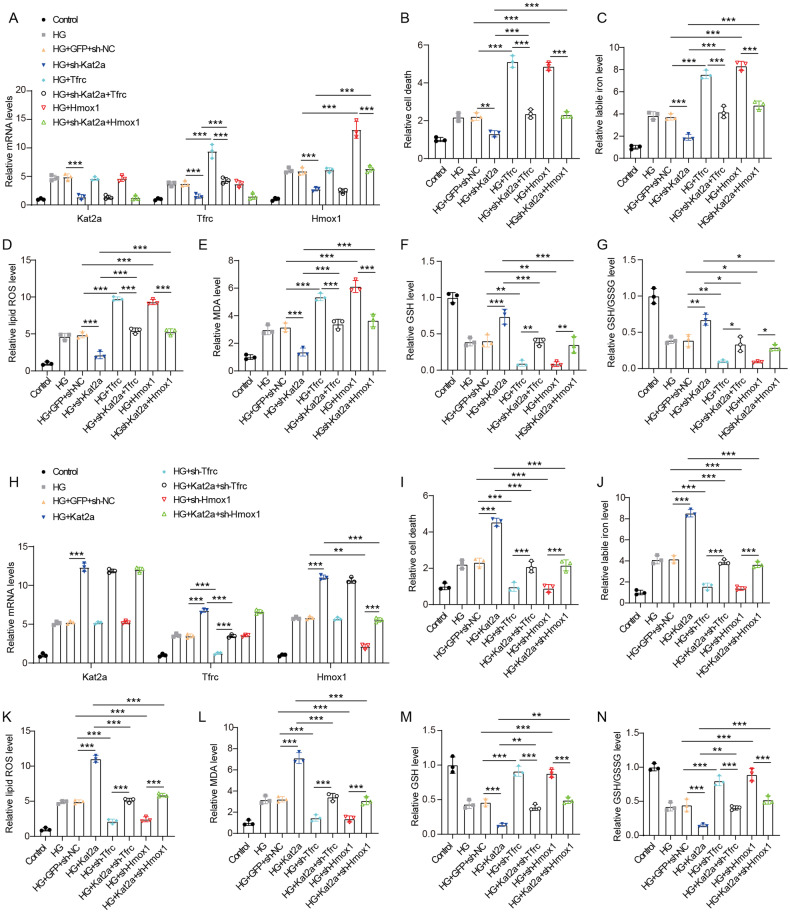
Kat2a aggravated ferroptosis by enhancing Tfrc and Hmox1 expression. **A** The mRNA expression levels of Kat2a, Tfrc, and Hmox1 were assessed in Kat2a-depleted NMVCs with overexpression of either Tfrc or Hmox1 (N = 3 independent experiments). **B** The cell death of Kat2a-depleted NMVCs with overexpression of either Tfrc or Hmox1 (N = 3 independent experiments). **C** The level of labile iron in Kat2a-depleted NMVCs with overexpression of either Tfrc or Hmox1 (N = 3 independent experiments). **D** The level of lipid ROS in Kat2a-depleted NMVCs with overexpression of either Tfrc or Hmox1 (N = 3 independent experiments). **E** The level of MDA in Kat2a-depleted NMVCs with overexpression of either Tfrc or Hmox1 (N = 3 independent experiments). **F**, **G** GSH and GSH/GSSG levels in Kat2a-depleted NMVCs with overexpression of either Tfrc or Hmox1 (N = 3 independent experiments). **H** The mRNA expression levels of Kat2a, Tfrc, and Hmox1 were assessed in Kat2a-overexpressing NMVCs with knockdown of either Tfrc or Hmox1 (N = 3 independent experiments). **I** The cell death of Kat2a-overexpressing NMVCs with knockdown of either Tfrc or Hmox1 (N = 3 independent experiments). **J** The level of labile iron in Kat2a-overexpressing NMVCs with knockdown of either Tfrc or Hmox1 (N = 3 independent experiments). **K** The level of lipid ROS in Kat2a-overexpressing NMVCs with knockdown of either Tfrc or Hmox1 (N = 3 independent experiments). **L** The level of MDA in Kat2a-overexpressing NMVCs with knockdown of either Tfrc or Hmox1 (N = 3 independent experiments). **M**, **N** GSH and GSH/GSSG levels in Kat2a-overexpressing NMVCs with knockdown of either Tfrc or Hmox1 (N = 3 independent experiments). Significance tested using One-way ANOVA. Statistical significance is shown as *p < 0.05, **p < 0.01, ***p < 0.001.

To verify whether Kat2a regulated ferroptosis via targeting Tfrc and Hmox1, we overexpressed Tfrc or Hmox1 in Kat2a-depleted NMVCs. Kat2a knockdown significantly reduced mRNA level of Kat2a, Tfrc, or Hmox1 in NMVCs treated with HG (Fig. 6A). Tfrc overexpression increased Tfrc expression and rescued the reduced Tfrc level induced by Kat2a knockdown, whereas it had no impact on Hmox1 expression. Hmox1 overexpression upregulated Hmox1 expression and reversed the decreased Hmox1 level induced by Kat2a knockdown, whereas it did not affect Tfrc expression. The HG-induced elevation in cell death, labile iron levels, lipid ROS production, and lipid peroxidation, as well as the decrease in GSH level and GSH/GSSG ratio were effectively rescued by Kat2a knockdown in NMVCs (Fig. 6B–G), indicating that Kat2a knockdown inhibited HG-induced ferroptosis. Overexpression of Tfrc resulted in upregulation of Tfrc mRNA expression in NMVCs under HG conditions, thereby inducing increased cell death, elevated levels of labile iron, lipid ROS and MDA, as well as reduced GSH and GSH/GSSG level. These findings suggested that Tfrc overexpression promoted ferroptosis in NMVCs under HG conditions. Besides, Tfrc overexpression rescued the inhibition effect of Kat2a knockdown on ferroptosis in NMVCs under HG conditions. Similarly, overexpression of Hmox1 was also observed to enhance ferroptosis in NMVCs under HG conditions and rescue the inhibitory effect of Kat2a knockdown on ferroptosis. Moreover, we also reduced Tfrc or Hmox1 expression in Kat2a-overexpressing NMVCs. Kat2a overexpression significantly increased the mRNA level of Kat2a, Tfrc, or Hmox1 in NMVCs under HG conditions (Fig. 6H). Tfrc knockdown reduced Tfrc mRNA expression and rescued the elevated Tfrc level induced by Kat2a overexpression. Consistently, Hmox1 knockdown reduced Hmox1 mRNA expression and rescued the elevated Hmox1 level induced by Kat2a overexpression. Kat2a overexpression promoted ferroptosis in NMVCs under HG conditions, whereas Tfrc or Hmox1 knockdown played opposite roles (Fig. 6I–N). Tfrc or Hmox1 knockdown rescued the accelerative effect of Kat2a overexpression on ferroptosis in NMVCs under HG conditions. In a word, our results suggest that Kat2a can regulate ferroptosis by enhancing Tfrc and Hmox1 expression.

### Kat2a deficiency suppresses DCM

**Fig. 7:**
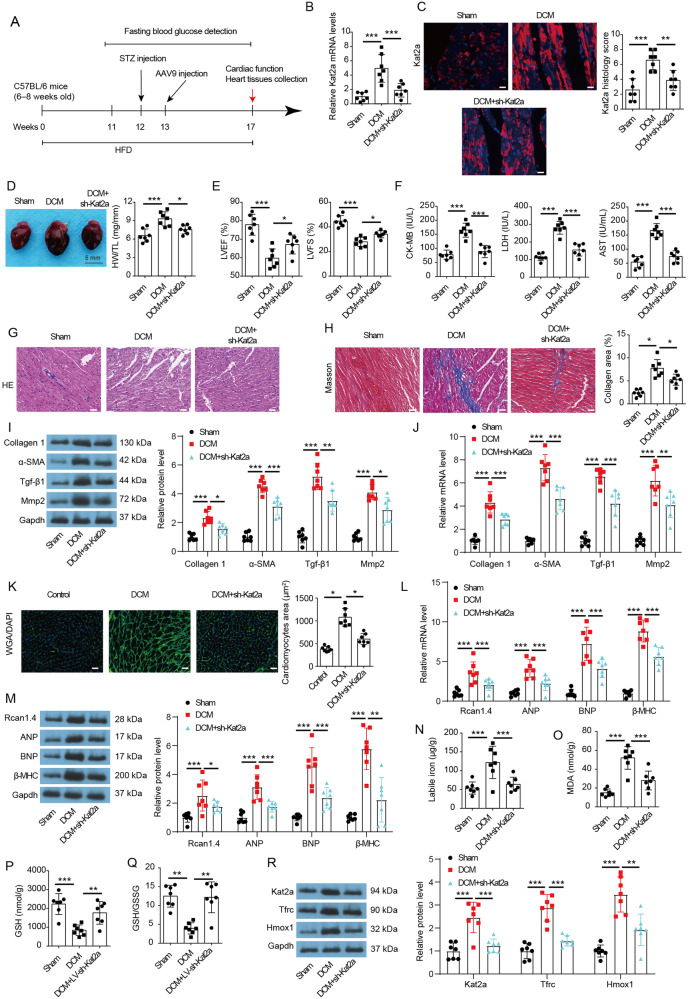
Kat2a deficiency ameliorated DCM-induced cardiac injury. **A** Schematic diagram of animal experiments. **B** Kat2a mRNA level in cardiac tissues (N = 7 mice/group). **C** Kat2a protein level in cardiac tissues was assessed by IF staining (N = 7 mice/group). Scale bar = 50 μm. **D** Representative images of cardiac specimens of each group and the ratio of heart weight to tibia length (HW/TL) were measured (N = 7 mice/group). Scale bar = 5 mm. **E** Quantitative analysis of LVEF and LVFS (N = 7 mice/group). **F** Serum CK-MB, LDH, and AST levels in mice (N = 7 mice/group). **G** Representative images of H&E staining of heart tissues. Scale bar = 50 μm. **H** Representative images of Masson staining of heart tissues and quantitative analysis of Collagen area (%) of Masson staining (N = 7 mice/group). Scale bar = 50 μm. **I** Protein levels of fibrosis markers (Collagen 1, α-SMA, Tgf-β1, and Mmp2) in heart tissue were detected by western blotting (N = 7 mice/group). **J** The mRNA expressions of fibrosis markers in heart tissue were detected by qPCR (N = 7 mice/group). **K** Representative images of wheat germ agglutinin (WGA) staining of heart tissues and quantitative analysis of cardiomyocytes area (µm^2^) of WGA staining (N = 7 mice/group). Scale bar = 50 μm. **L** The mRNA expressions of hypertrophic markers (Rcan1.4, ANP, BNP, and β-MHC) in heart tissue were detected by qPCR (N = 7 mice/group). **M** Protein levels of hypertrophic markers in heart tissue were detected by western blotting (N = 7 mice/group). **N**–**Q** The level of labile iron, MDA, GSH, and GSH/GSSG in heart tissues was measured (N = 7 mice/group). **R** Protein levels of Kat2a, Tfrc, and Hmox1 in heart tissue were detected by western blotting (N = 7 mice/group). Significance tested using One-way ANOVA. Statistical significance is shown as *p < 0.05, **p < 0.01, ***p < 0.001.

To explore the role of Kat2a in DCM, AAV9 was applied via tail vein injection to reduce the expression profile of Kat2a in vivo (Fig. 7A). AAV-sh-Kat2a significantly inhibited Kat2a mRNA and protein expression in cardiac tissues of DCM mice (Fig. 7B, C). The DCM model was established, and cardiac function and the extent of myocardial injury were analyzed. The heart of the DCM group was larger and the enlarged heart phenotype was ameliorated in the Kat2a deficiency group (Fig. 7D). The ratio of heart weight to tibial length (HW/TL) was increased in the DCM group, while Kat2a deficiency improved this change. Echocardiography examination revealed a reduction in LVEF and LVFS ratio in DCM mice, implying impaired heart failure of DCM mice (Fig. 7E). Kat2a deficiency increased LVEF and LVFS in DCM mice, suggesting that Kat2a knockdown relieved heart failure in DCM mice. To further assess cardiac injuries, various injury-related biomarkers including CK-MB, LDH, and AST were detected (Fig. 7F). Kat2a knockdown restored the DCM-induced increase of serum CK-MB, LDH, and AST level. These data indicated that Kat2a deficiency ameliorated DCM-induced cardiac injury. HE staining of heart tissues from DCM mice revealed an enlarged morphology and disorganized arrangement of cardiac cells, associated with inflammatory cell filtration. However, these abnormal changes were improved after Kat2a knockdown (Fig. 7G). The fibrotic state of heart tissues was assessed by Masson staining (Fig. 7H). DCM could result in severe cardiac fibrosis in mice, which was significantly reduced upon Kat2a knockdown, as evidenced by decreased collagen deposition. Moreover, the enhanced fibrosis in DCM mice was also confirmed by higher Collagen 1, α-SMA, Tgf-β1, and Mmp2 protein and mRNA expression in myocardial tissue (Fig. 7I, J). Importantly, Kat2a knockdown rescued these pro-fibrotic gene expressions in myocardial tissue, indicating that Kat2a knockdown improved myocardial fibrosis induced by DCM. Additionally, Wheat germ agglutinin (WGA) staining indicated that the cardiomyocyte size was increased in DCM mice and Kat2a knockdown could improve this change (Fig. 7K). We also analyzed the mRNA and protein expression of hypertrophic markers in myocardial tissue, including regulator of Calcineurin 1 Isoform 4 (Rcan1.4), brain natriuretic peptide (BNP), atrial natriuretic peptide (ANP), and beta-myosin heavy chain (β-MHC). The myocardial tissue of DCM mice showed higher expression of these hypertrophic markers (Fig. 7L, M). Kat2a knockdown reversed the upregulation of these pro-hypertrophic markers in myocardial tissue from DCM mice, indicating that Kat2a knockdown improved cardiac hypertrophy induced by DCM. Moreover, we observed an elevation in cardiac labile iron and MDA, as well as a reduction in GSH level and GSH/GSSG ratio in DCM mice (Fig. 7N–Q). Notably, elevated protein expression of key regulators of ferroptosis (Tfrc and Hmox1) also were found in myocardial tissue in DCM mice (Fig. 7R). Kat2a deficiency damped these changes, suggesting that Kat2a deficiency inhibited ferroptosis in DCM mice. Overall, our findings provide compelling evidence for the preventive role of Kat2a deficiency against DCM.

### Kat2a regulates inflammation in DCM

**Fig. 8:**
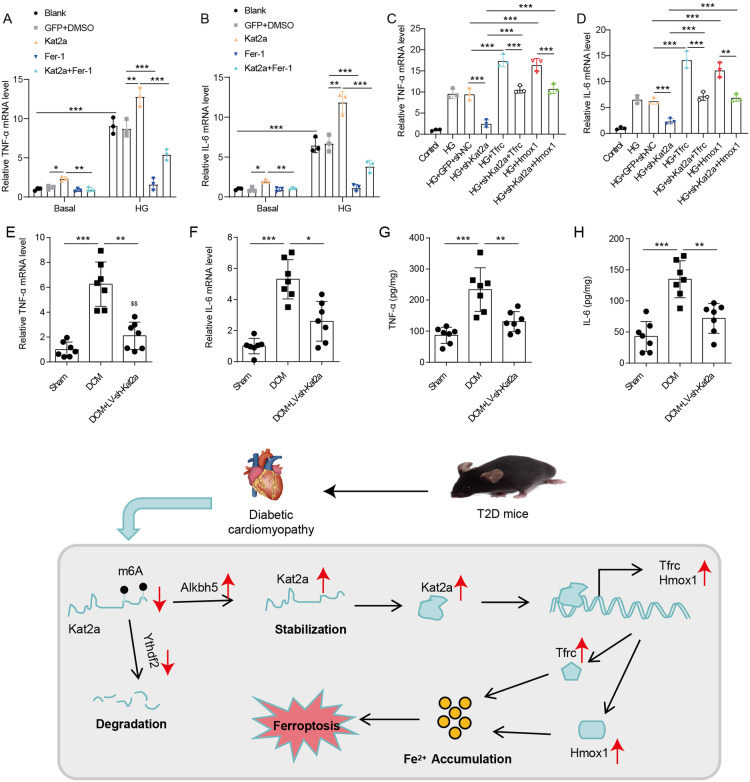
Kat2a deficiency protected DCM from inflammatory injury by inhibiting ferroptosis. **A**, **B** TNF-α and IL-6 mRNA levels in NMVCs with Kat2a overexpression or Fer-1 treatment under basal or HG conditions (N = 3 independent experiments). **C**, **D** TNF-α and IL-6 mRNA levels in NMVCs with Kat2a knockdown along with Tfrc or Hmox1 overexpression (N = 3 independent experiments). **E,** **F** TNF-α and IL-6 mRNA levels in cardiac tissues of mice (N = 7 mice/group). **G**, **H** TNF-α and IL-6 protein levels in the cardiac tissues of mice were measured by ELISA (N = 7 mice/group). **I** Graphical summary of the mechanistic study of this study. Significance tested using One-way ANOVA. Statistical significance is shown as *p < 0.05, **p < 0.01, ***p < 0.001.

As inflammation is a key pathogenic feature of DCM, we then explored the effect of Kat2a on inflammation. The mRNA levels of TNF-α and IL-6 were markedly increased in NMVCs treated with HG (Fig. 8A, B). Under basal conditions, overexpression of Kat2a significantly upregulated the mRNA expression levels of TNF-α and IL-6, while treatment with Fer-1 had minimal impact on the expression of these cytokines. Remarkably, Fer-1 treatment effectively reversed the elevated levels of TNF-α and IL-6 induced by Kat2a overexpression. Under HG conditions, Kat2a overexpression led to an increase in TNF-α and IL-6 mRNA expression, whereas Fer-1 treatment attenuated their expression. Furthermore, Fer-1 treatment substantially rescued the enhanced expression of TNF-α and IL-6 caused by Kat2a overexpression. In addition, Kat2a knockdown reduced TNF-α and IL-6 expression in NMVCs treated with HG (Fig. 8C, D), while Tfrc or Hmox1 overexpression increased their expression. The overexpression of Tfrc or Hmox1 rescued the decreased TNF-α and IL-6 expression induced by Kat2a knockdown, indicating that Kat2a could affect inflammation of HG-treated NMVCs by targeting Tfrc or Hmox1. Besides, DCM induction increased the mRNA and protein expression of TNF-α and IL-6 in cardiac tissues, which were significantly inhibited by Kat2a deficiency (Fig. 8E–H). These findings demonstrate that Kat2a deficiency confers protection against inflammatory injury in DCM.

## Discussion

Mechanistic studies are crucial for improving prevention and treatment strategies for DCM. In our present study, we first revealed the involvement of Kat2a in DCM by showing a significant increase in Kat2a level both within HG-treated myocardial cells and cardiac tissues affected by DCM. The upregulation of Kat2a was facilitated by the enhanced Alkbh5-induced demethylation and attenuated Ythdf2-dependent degradation. Furthermore, we found that Kat2a played a crucial role in the epigenetic activation of Tfrc and Hmox1 transcription, thereby facilitating the process of ferroptosis. Reduction of cardiac Kat2a inhibited ferroptosis and inflammation, leading to the amelioration of DCM (Fig. 8I). These findings highlight the crucial function of the cardiac Kat2a-ferroptosis axis in DCM progression.

m6A is a prevalent mRNA epigenetic regulation that affects various biological processes by regulating the expression of target genes [19]. The m6A regulatory pathway consists of m6A writer-complex components, m6A readers (YTHDFs and IGF2BPs), as well as potential erasers (FTO and ALKBH5) [20]. Furthermore, m6A has been implicated in myocardial ischemia-reperfusion injury [21], cardiomyopathy [22], and DCM [23]. METTL14-mediated m6A methylation decreases TINCR expression, leading to the inhibition of NLRP3-induced pyroptosis and amelioration of DCM [24]. Peng et al. indicate that Airn inhibits cardiac fibrosis in an m6A-IMP2-p53 axis [25]. Moreover, YTHDC1 inhibition promotes the development of dilated cardiomyopathy [26]. Herein, we conducted a screening of abnormally expressed mRNAs with modified m6As in heart tissues and identified a significant decrease in the levels of modified Kat2a. Mechanistically, we found that Kat2a expression in DCM was tightly controlled by its m6A modification. Demethylase Aklbh5 reduced the level of Kat2a mRNA’s m6A modification state resulting in its upregulation. m6A reader Ythdf2 was essential for mediating the degradation of Kat2a mRNA. Moreover, we observed upregulation of Aklbh5 along with downregulation of YTHDF2 within heart tissues affected by DCM; this further deteriorates the condition via an Aklbh5-m6A-Ythdf2 axis inducing upregulation of Kat2a mRNA expression levels. Our study revealed the mode of m6A in DCM, which may be improved by regulating the m6A modification state of kat2a.

Ferroptosis is a form of programmed cell death that relies on iron-catalyzed lipid peroxidation, distinguishing it from autophagy, apoptosis, and necrosis [27]. Numerous studies have demonstrated the involvement of ferroptosis in myocardial injury, including DCM [11, 28]. Our data further substantiate the association between ferroptosis and DCM. Through bioinformatics analysis, we identified upregulated expression of genes involved in ferroptosis, such as Tfrc and Hmox1, in the heart tissue of DCM mice. Tfrc overexpression promotes ferroptosis in CVB3 infection-induced myocarditis [29] and doxorubicin-induced cardiomyocyte [22]. Hmox1 upregulation facilitates ferroptosis in diabetic atherosclerosis [30], diabetic nephropathy [31], myocardial ischemia reperfusion injury [32], and cardiomyopathy [33]. In this study, we discovered that Kat2a epigenetically activated the transcription of Tfrc and Hmox1. By increasing Tfrc and Hmox1 expression levels, Kat2a promoted iron accumulation and lipid peroxidation in HG-treated myocardial cells. Depletion of Kat2a inhibited ferroptosis and alleviated diabetes mellitus-induced cardiac damage. Based on these findings, we conclude that inhibition of ferroptosis through cardiac kat2a depletion improves DCM.

Inflammation is a pivotal pathogenic feature associated with various diseases including DCM. Hyperglycemia and increased metabolism of free fatty acids are linked to upregulated proinflammatory cytokines such as IL-6 and TNF-α [34]. The inflammatory response plays a role in regulating myocardial cell death, while promoting fibrosis and hypertrophy, ultimately leading to abnormal contractility of myocardial cells which contributes to the development of DCM [1]. Therefore, potential therapeutic strategies targeting inflammation may be expected to improve DCM. Here, we confirmed the upregulation of proinflammatory cytokines IL-6 and TNF-α in cardiac tissues of DCM mice and high glucose-treated myocardial cells. Kat2a promotes inflammation in high glucose-treated myocardial cells by activating ferroptosis. Cardiac depletion of Kat2a significantly reduces DCM-induced levels of IL-6 and TNF-α, indicating that Kat2a depletion ameliorates DCM by inhibiting inflammation.

The release of myocardial enzymes into the serum serves as an index for diagnosing and monitoring myocardial injury when the myocardium is damaged or dies. Consistent with previous studies on DCM [35, 36], our results demonstrated a significant elevation in AST, LDH, and CK-MB levels in DCM. Moreover, the depletion of Kat2a led to a significant reduction in these markers of myocardial injury, indicating that it protects the integrity of the cell membrane and prevents enzyme leakage. In summary, our findings reveal a novel role for the Kat2a-ferroptosis axis in DCM pathogenesis and provide a potential target for clinical treatment.

In conclusion, we found the m6A modification of Kat2a is important for the development of DCM and displayed a novel role of the Kat2a-ferroptosis axis in DCM pathogenesis and provides a potential target for clinical treatment.

### Supplementary information

Full and uncropped western blots

Supplementary materials

## Data Availability

The datasets used and/or analyzed during the current study are available from the corresponding author upon reasonable request.
