# Supplementary material for: Histone acetyltransferase Kat2a regulates ferroptosis via enhancing Tfrc and Hmox1 expression in diabetic cardiomyopathy
Source: Cell Death Dis. 2024 Jun 10;15(6):406. doi: 10.1038/s41419-024-06771-x (PMC11164963; doi:10.1038/s41419-024-06771-x)
Supplement: Supplementary file 1 — Full and uncropped western blots [file 41419_2024_6771_MOESM1_ESM.docx]

**Fig 1H**

**
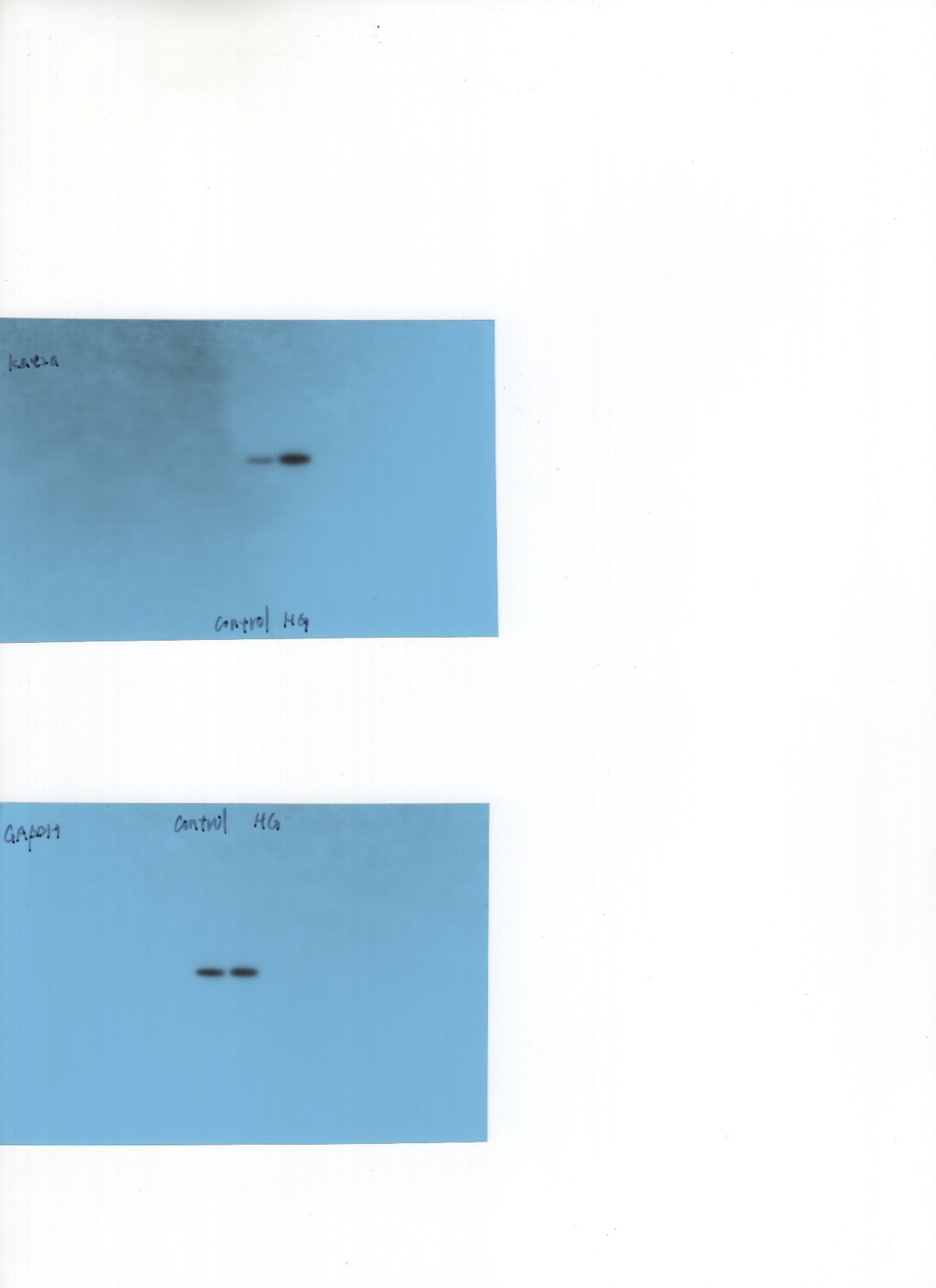
**

94 kDa

37 kDa

**Fig 2D**

**
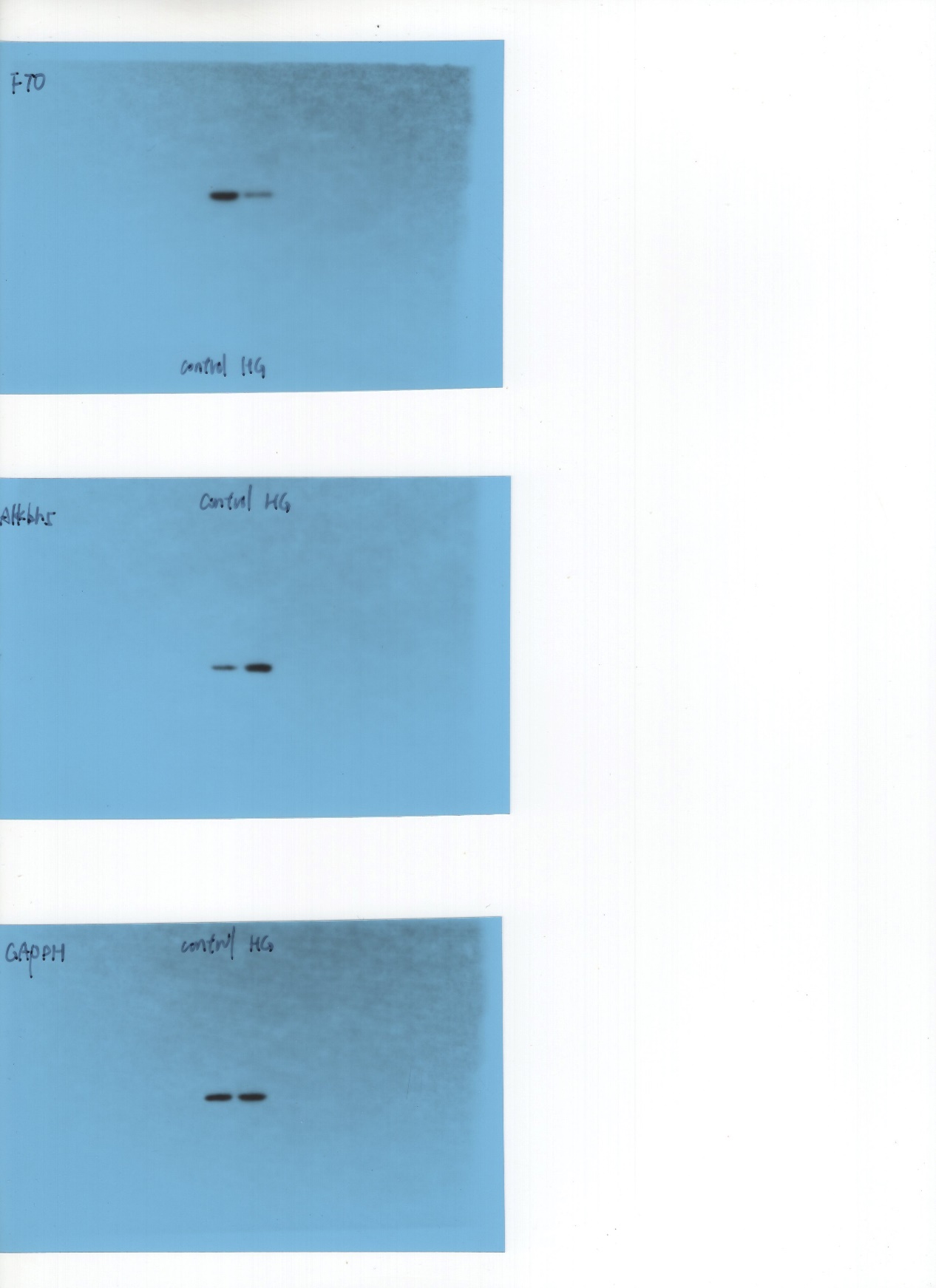
**

37 kDa

48 kDa

58 kDa

**Fig 2G**

**
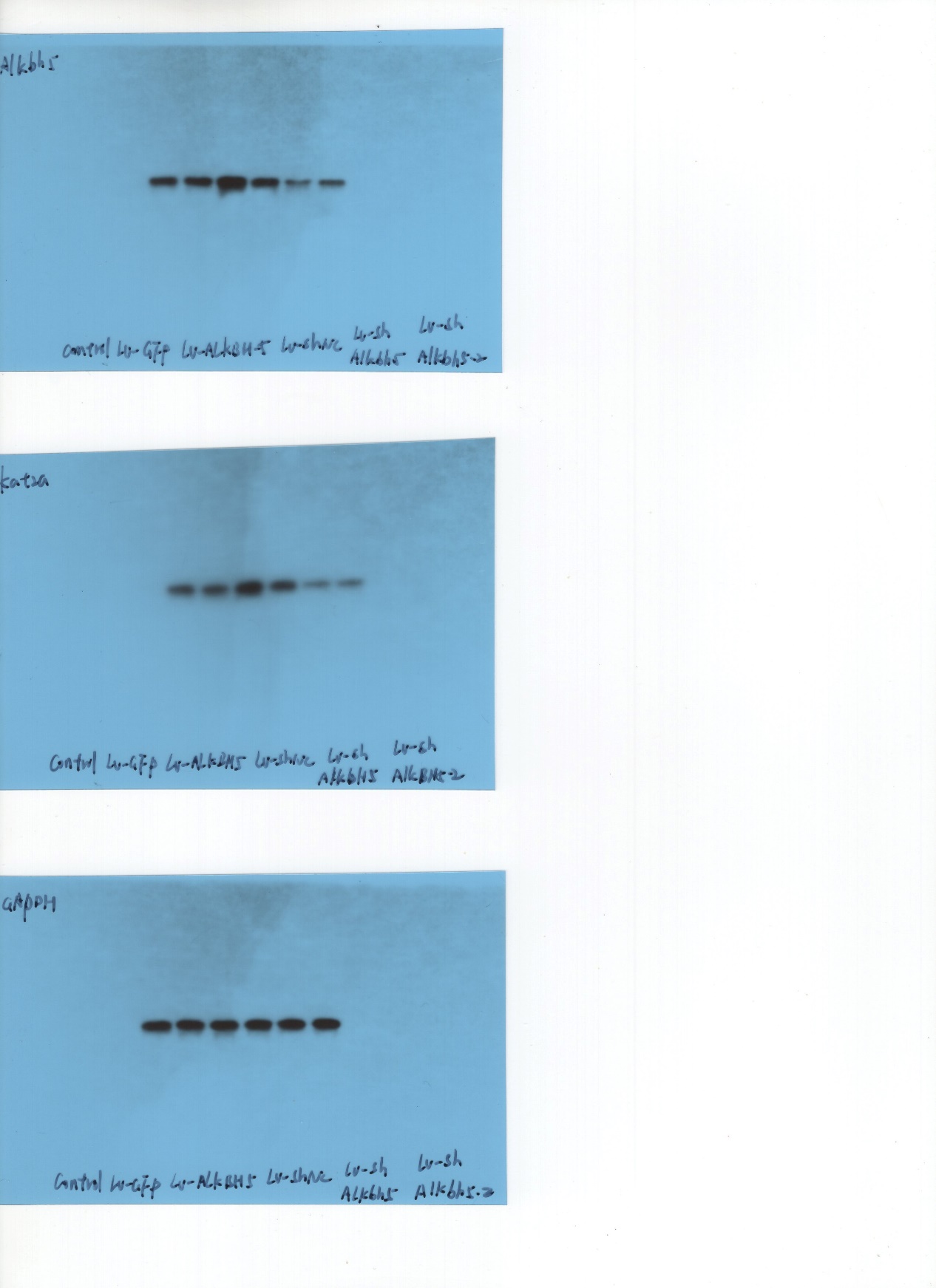
**

37 kDa

94 kDa

48 kDa

**Fig 3A**

**
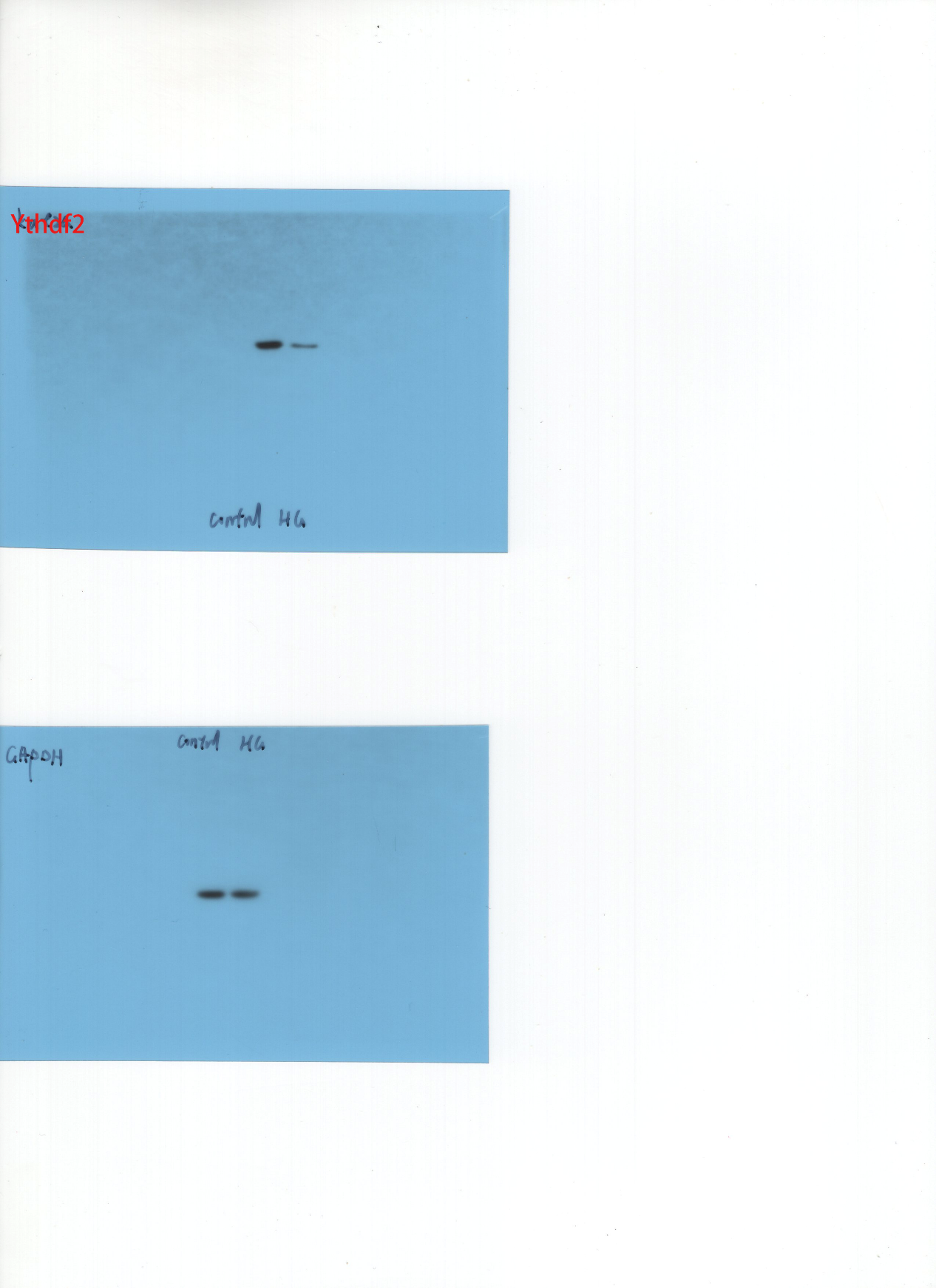
**

37 kDa

62 kDa

**Fig 3E**

**
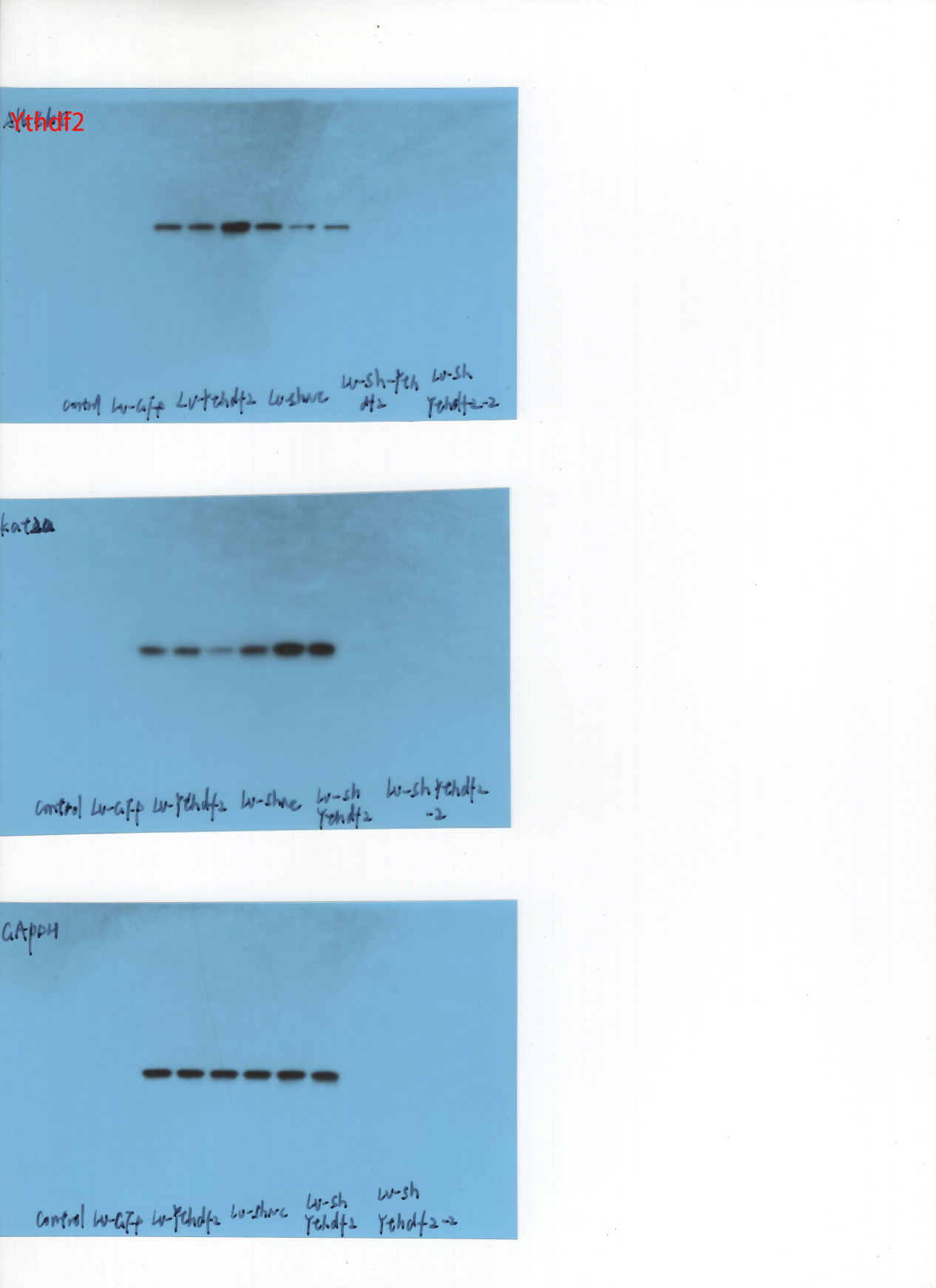
**

37 kDa

94 kDa

62 kDa

**Fig 7I**

**
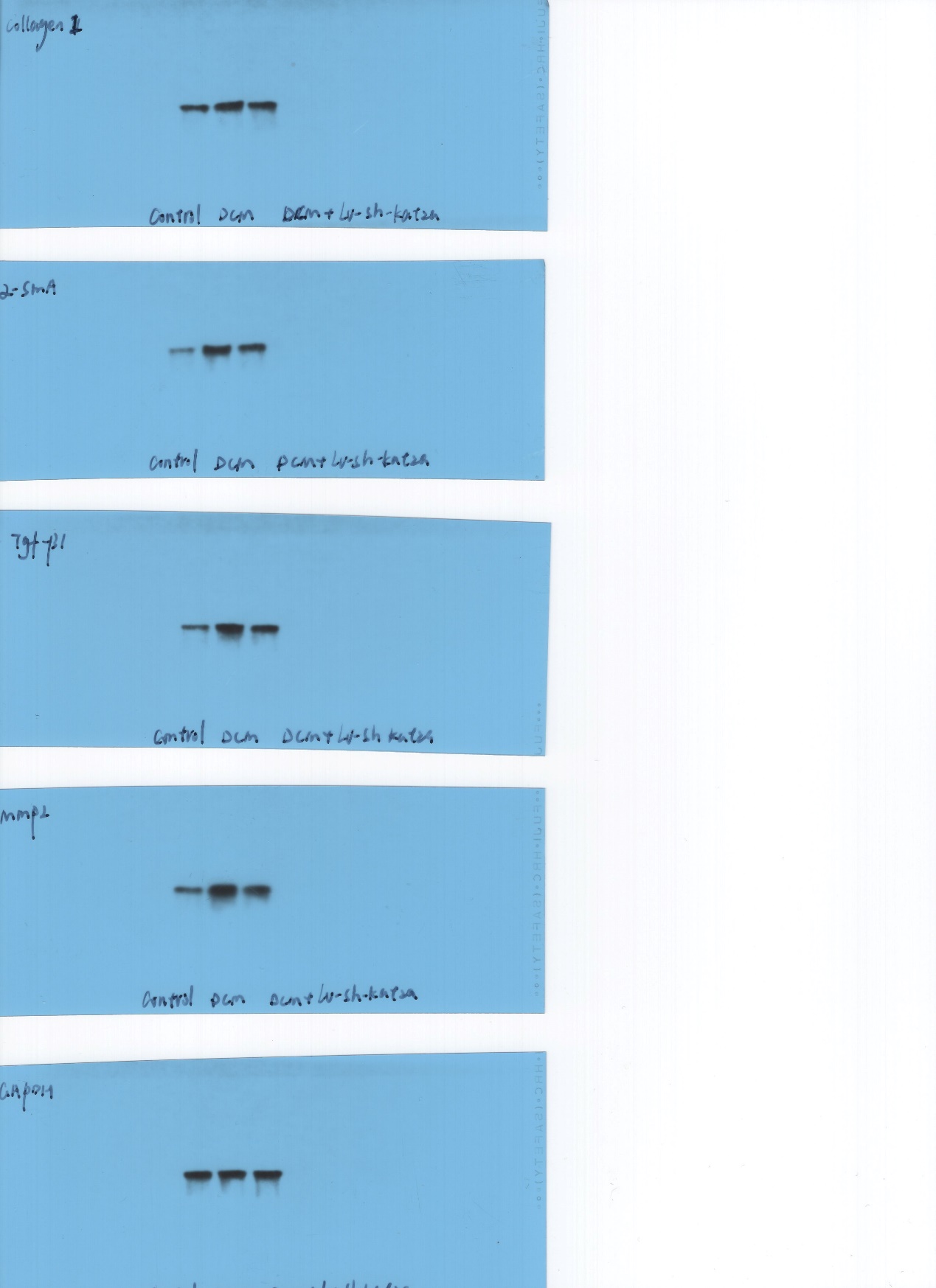
**

37 kDa

72 kDa

44 kDa

42 kDa

130 kDa

**Fig 7M**

**
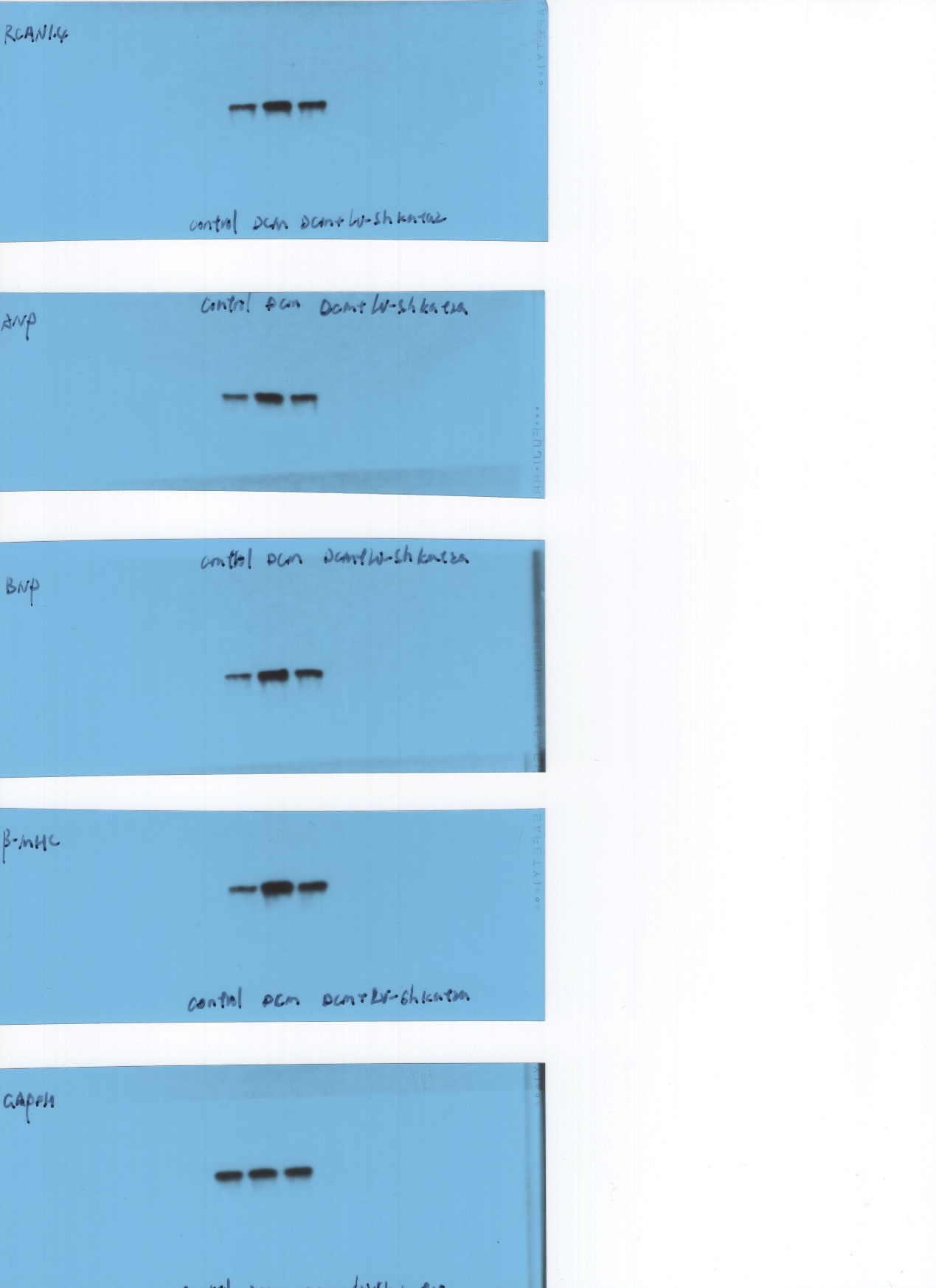
**

37 kDa

200 kDa

17 kDa

17 kDa

28 kDa

**Fig 7R**

**
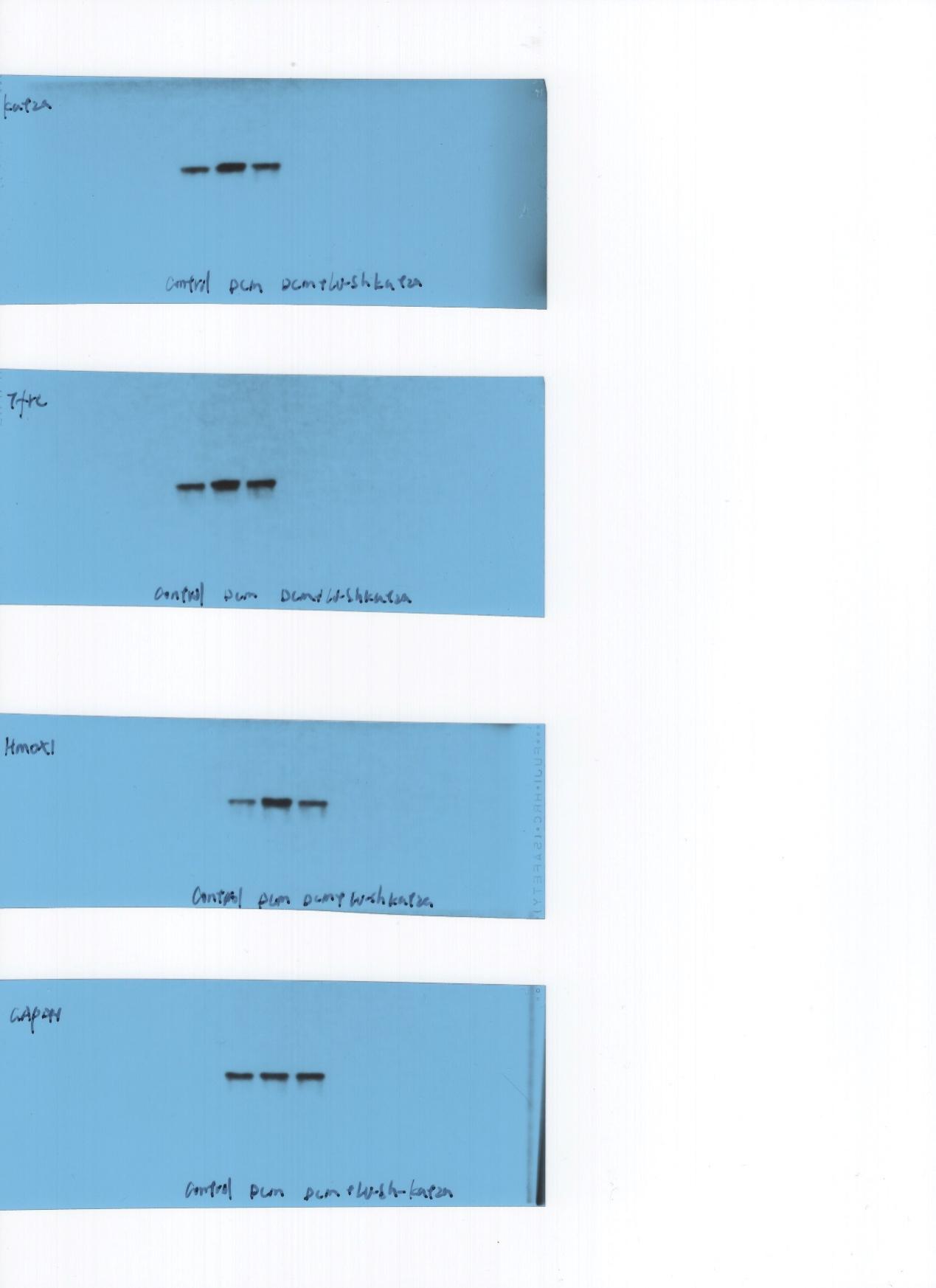
**

37 kDa

32 kDa

90 kDa

94 kDa
