# Supplementary material for: Histone acetyltransferase Kat2a regulates ferroptosis via enhancing Tfrc and Hmox1 expression in diabetic cardiomyopathy
Source: Cell Death Dis. 2024 Jun 10;15(6):406. doi: 10.1038/s41419-024-06771-x (PMC11164963; doi:10.1038/s41419-024-06771-x)
Supplement: Supplementary file 2 — Supplementary materials [file 41419_2024_6771_MOESM2_ESM.docx]

**Supplementary Table S1 The sequence of shRNA used in this study**

| **ID** | **Sequence 5’−3’** |
| --- | --- |
| sh-NC | TTCTCCGAACGTGTCACGT |
| sh-Alkbh5-1 | GGGAGAAGCTCAAGTCCATGA |
| sh-Alkbh5-2 | GCATACGGCCTCAGGACATTA |
| sh-Ythdf2-1 | GGTCCATCACTAGTAACATTG |
| sh-Ythdf2-2 | GCACAGAGCATGGTAACAAGA |
| sh-Kat2a-1 | GGACACAGACACCAAACAAGT |
| sh-Kat2a-2 | GCCAAGAATGCCCAAGGAATA |
| sh-Tfrc- | TTTAATAACTCCAAAGATGTT |
| sh-Hmox1 | CCACACAGCACTATGTAAATT |

**Supplementary Table S2 The primers used in qRT-PCR**

| **Primer** | **Sequence 5’−3’** |
| --- | --- |
| Kat2a sense | CAGATCCGCAAGGTCTACCC |
| Kat2a anti-sense | TCCAGCCTGTCTCTCGAATG |
| IL-6 sense | GCACTAGGTTTGCCGAGTAGA |
| IL-6 anti-sense | GAGGAAGACACTGAGGTCGAA |
| TNF-α sense | ATCCGCGACGTGGAACTAG |
| TNF-α anti-sense | AAGGTCTTGAGGTCCGCCA |
| Gapdh sense | AGAACATCATCCCTGCATCC |
| Gapdh anti-sense | AGTTGCTGTTGAAGTCGC |
| Hmox1 sense | GAGACGGCTTCAAGCTGGTGAT |
| Hmox1 anti-sense | CCGTACCAGAAGGCCAGGTC |
| Tfrc sense | CCCCTTTACAATAGCCCAAG |
| Tfrc anti-sense | ACCTGGTGTTACCCACTTTCC |
| ANP sense | ACCTGCTAGACCACCTGGAG |
| ANP anti-sense | CCTTGGCTGTTATCTTCGGTACCGG |
| BNP sense | GAGGTCACTCCTATCCTCTGG |
| BNP anti-sense | GCCATTTCCTCCGACTTTTCTC |
| β-MHC sense | CGGACCTTGGAAGACCAGAT |
| β-MHC anti-sense | GACAGCTCCCCATTCTCTGT |
| Collagen 1 sense | AGGCTTCAGTGGTTTGGATG |
| Collagen 1 anti-sense | CACCAACAGCACCATCGTTA |
| Rcan1.4 sense | CCCGTGAAAAAGCAGAATGC |
| Rcan1.4 anti-sense | TCCTTGTCATATGTTCTGAAGAGGG |
| Mmp2 sense | TTCCCTAAGCTCATCGCAGACT |
| Mmp2 anti-sense | CACGCTCTTGAGACTTTGGTTCT |
| α-SMA sense | CAGGATGCAGAAGGAGATCA |
| α-SMA anti-sense | TCCACATCTGCTGGAAGGTA |
| Tgf-β1 sense | CAACAATTCCTGGCGTTACCTTGG |
| Tgf-β1 anti-sense | GAAAGCCCTGTATTCCGTCTCCTT |
